# A comprehensive review of heregulins, HER3, and HER4 as potential therapeutic targets in cancer

**DOI:** 10.18632/oncotarget.18467

**Published:** 2017-06-13

**Authors:** Jose Mauricio Mota, Katharine Ann Collier, Ricardo Lima Barros Costa, Timothy Taxter, Aparna Kalyan, Caio A. Leite, Young Kwang Chae, Francis J. Giles, Benedito A. Carneiro

**Affiliations:** ^1^ Instituto do Câncer do Estado de São Paulo, Division of Oncology, Universidade de São Paulo, São Paulo, Brazil; ^2^ Developmental Therapeutics Program, Division of Hematology and Oncology, Feinberg School of Medicine, Northwestern University, Chicago, Illinois, USA; ^3^ Department of Medicine, Feinberg School of Medicine, Northwestern University, Chicago, Illinois, USA; ^4^ Department of Pharmacology, Ribeirão Preto Medical School, University of São Paulo, São Paulo, Brazil

**Keywords:** heregulins, HER3, HER4, cancer, developmental therapeutics

## Abstract

Heregulins (HRGs) bind to the receptors HER3 or HER4, induce receptor dimerization, and trigger downstream signaling that leads to tumor progression and resistance to targeted therapies. Increased expression of HRGs has been associated with worse clinical prognosis; therefore, attempts to block HRG-dependent tumor growth have been pursued. This manuscript summarizes the function and signaling of HRGs and review the preclinical evidence of its involvement in carcinogenesis, prognosis, and treatment resistance in several malignancies such as colorectal cancer, non-small cell lung cancer, ovarian cancer, and breast cancer. Agents in preclinical development and clinical trials of novel therapeutics targeting HRG-dependent signaling are also discussed, including anti-HER3 and -HER4 antibodies, anti-metalloproteinase agents, and HRG fusion proteins. Although several trials have indicated an acceptable safety profile, translating preclinical findings into clinical practice remains a challenge in this field, possibly due to the complexity of downstream signaling and patterns of HRG, HER3 and HER4 expression in different cancer subtypes. Improving patient selection through biomarkers and understanding the resistance mechanisms may translate into significant clinical benefits in the near future.

## INTRODUCTION

Heregulins (HRGs; aliases: neuregulin, Neu differentiation factor, glial growth factor, acetylcholine receptor-inducing activity) are growth factors that trigger multi-step kinase-dependent signaling events after binding to the transmembrane receptors HER3 or HER4. Deregulation of this pathway have been linked to several conditions including schizophrenia, heart failure, atherosclerosis, and cancer. Specifically in cancer, HRGs are implicated in stemness, invasiveness, proliferation, resistance to apoptosis, and angiogenesis.[1, 2] This manuscript summarizes the role of HRGs in carcinogenesis and advances in the development of novel therapies targeting HRG-mediated pathways.

## HEREGULIN STRUCTURE AND SIGNALING MECHANISM

A 44-kDa glycoprotein, later to be classified as an HRG, was isolated and cloned from RAS-transformed rat fibroblasts in 1992.[3] The protein named Neu differentiation factor induced phosphorylation of p185^neu^ (later called HER2/neu) and differentiation of human breast cancer cells.[3] Almost concomitantly, another group purified and cloned a 45-kDa protein from a human breast cell line that induced phosphorylation of p185^neu^/HER2, which they named HRG.[4]

Four major types of HRG proteins have been described: HRG-1 (subdivided in type I, II and III), HRG-2, HRG-3 and HRG-4. Alternate splicing of the four genes results in at least 26 different isoforms with distinct binding affinities to the HER family of receptors.[1] The protein structure is comprised of an N-terminus motif, an Ig-like domain (HRG-1 types I and II and HRG-2), an EGF-like domain, a juxtamembrane domain, a transmembrane domain, and a cytoplasmic tail. The proteins are synthesized as large membrane-anchored glycosylated precursors, with the EGFR-like motif positioned in the extracellular compartment.

HRGs were thought to be HER2 ligands since they cause phosphorylation of HER2. However, they were incapable of stimulating tyrosine kinase phosphorylation in fibroblasts overexpressing HER2.[5] Rather, it was shown that the HRGs interact with HER3 and HER4, but do not bind to HER2 receptors.[6] The extracellular EGF-like domain of HRG is essential for binding to and activation of HER3 in a juxtacrine fashion.[7] Furthermore, the HRG protein can be cleaved from the cellular membrane by metalloproteinases and result in paracrine or autocrine signaling.[8]

When HRG binds to HER3 or HER4, the dimerization arm is untethered, resulting in heterodimerization or homodimerization of HER3 or HER4 with HER4 or, preferentially, HER2.[9] HER3 does not homodimerize after HRG binding. Receptor dimerization activates tyrosine kinase activity, leading to trans-phosphorylation of the tyrosine-rich C-terminal region of HER3 or auto-phosphorylation of HER4. [10–13] In the case of HER3/HER2 dimerization, HER3, which is a kinase-dead receptor, does not phosphorylate HER2; rather, the dimerization results in a conformational change in HER2 resulting in activation of its downstream signaling. [14] The C-terminal phosphorylation motifs depend on the ligand isoform and different dimer combinations and, in turn, elicit different downstream signaling events. Therefore, the predominant types of HER present on the cell surface, in addition to the HRG isoform, affect downstream signaling that result in cell migration, proliferation, differentiation or apoptosis.[15]

**Figure 1:**
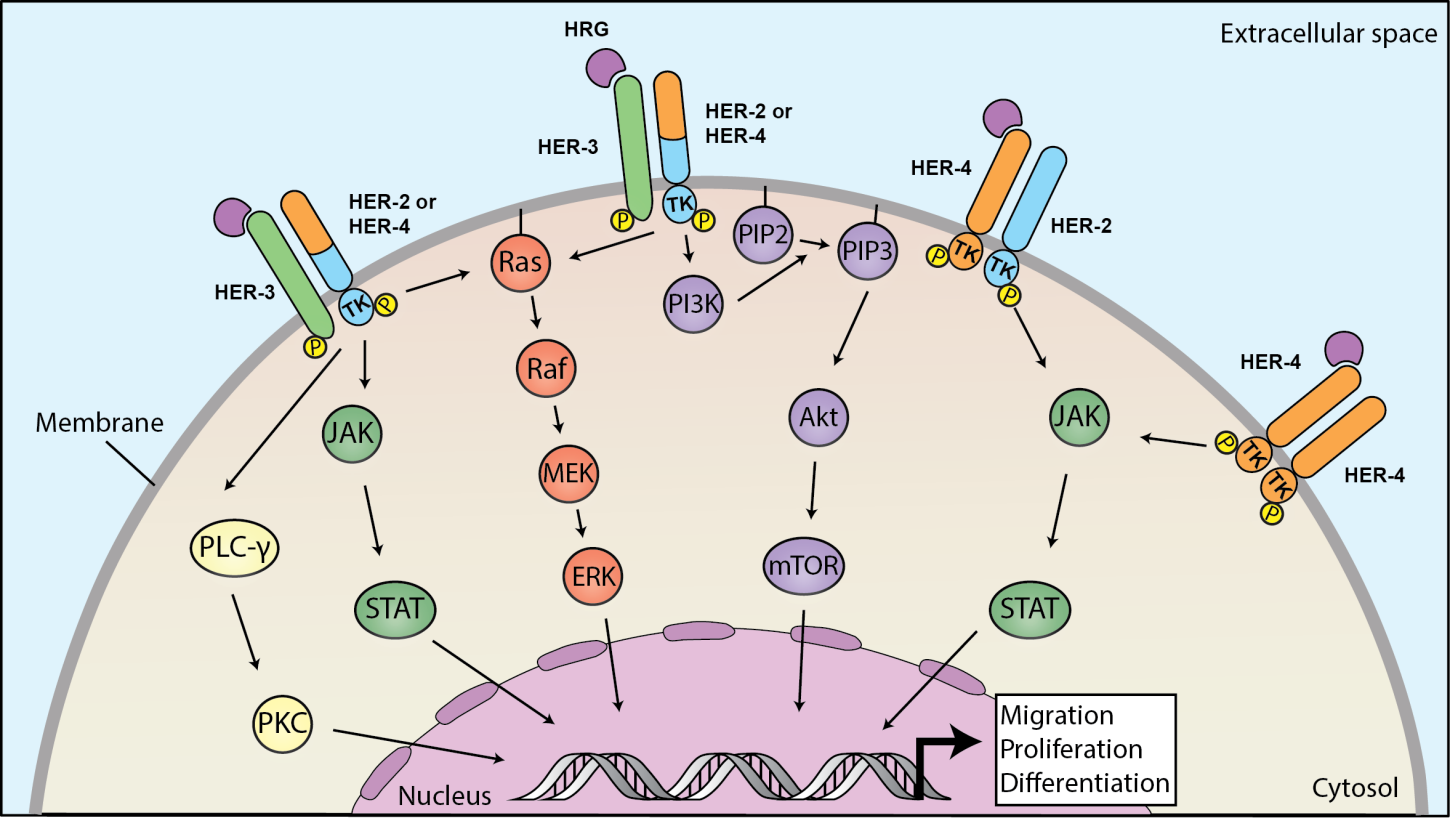
Heregulins bind to HER3 or HER4 to mediate downstream signaling linked to carcinogenesis Binding of heregulin HER3 or HER4 triggers dimerization to HER2 or HER4 and phosphorylation of intracellular domains, leading to activation of downstream pathways. HER3-activated pathways involve MAPK/ERK, PI3K/AKT/MTOR, JAK/STAT and PKC and HER4 that promote proliferation, migration and differentiation of cancer cell. HER: human epithelial growth factor receptor; MAPK: mitogen-activated protein kinase; ERK: extracellular signal-regulated kinases; PI3K: phosphoinositide 3-kinase; AKT: protein kinase B; MTOR: mechanistic target of rapamycin; JAK: Janus kinase; STAT: signal transducer and activator of transcription; PKC: protein kinase C.

Figure 1 depicts the how HRG elicits several intracellular pathways after binding to HER3 or HER4. HER3-mediated signaling involves the MAPK/ERK, PI3K/AKT/MTOR, JAK/STAT, and PKC protein kinase pathways.[16] In general, HER3-containing heterodimers (i.e. HER2/HER3 and HER3/HER4) are tumor-promoting.[17] On the other hand, HER4-mediated signaling results in either pro-tumor or anti-tumor effects through multiple protein kinase pathways, including JAK/STAT and PI3K/AKT. HER4 activating mutations upregulate the PI3K/AKT pathway.[18] In cancer cells, HER4 promotes proliferation, invasion, and cell migration, or differentiation and apoptosis. [19–24] When HRG1 binds to HER4, juxtamembrane and intramembrane proteolysis causes release of a soluble intracellular domain, which relocates to the nucleus, activates YAP, and mediates transcription of YAP/HIPPO target genes involved with proliferation and apoptosis.[22]

**Figure 2:**
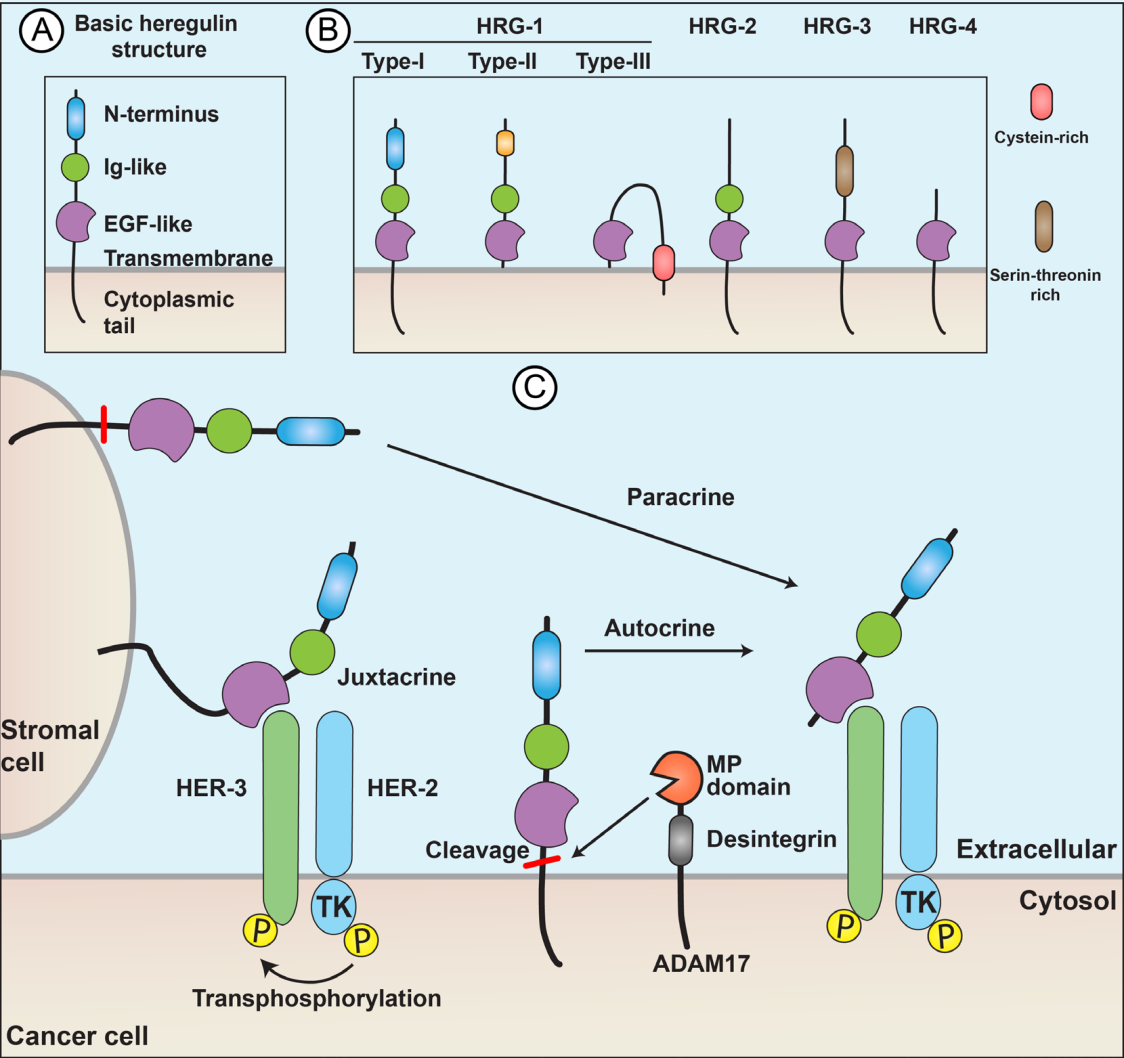
Heregulins act through paracrine, juxtacrine, or autocrine signaling (**A**) The protein structure includes an N-terminus motif, an Ig-like domain (HRG-1 types I and II and HRG-2), an EGF-like domain a juxtamembrane domain a transmembrane domain (the most variable region among different HRG subtypes) and a cytoplasmic tail. (**B**) Basic structural differences of HRG subtypes. The EGF-like domain is highly conserved across species and is essential and sufficient for receptor binding and activation. (**C**) Paracrine, juxtacrine, and autocrine signaling. Autocrine and paracrine signaling depend on metalloproteinases, such as ADAM17, which cleave the HRG from cell membrane.

Regulation of the HRG/HER signaling pathway occurs at many points. Competitive receptors such as p85-soluble-ErbB3 can trap HRG and prevent activation of HER2, HER3 and HER4.[25] In the case of or autocrine signaling, cleavage of the extracellular EGF-like domain from the HRG protein is dependent on metalloproteinases. Figure 2 illustrates how HRGs act in paracrine, juxtacrine and autocrine ways. The presence and activity of the metalloproteinases in turn control the amount of released HRG protein available to bind to HER receptors. For example, ADAM17, a disintegrin and metalloproteinase 17, mediates motility and angiogenesis associated with colon cancer cells through neuregulin-1.[26] Any process modulating the expression of HER2, HER3, and HER4 on the cell surface alter the effect of HRG/HER signaling. For example, NEDD4 (neural precursor cell expressed developmentally downregulated-4), an E3 ubiquitin ligase, can reduce HER3 expression on the cell surface.[27]

## THE HEREGULINS-DEPENDENT PATHWAY IN DIFFERENT CANCER TYPES

### Colorectal cancer (CRC)

HRGs, HER3, and HER4 are implicated in CRC carcinogenesis. HRGs contribute to colonic carcinogenesis through increased cyclooxygenase-2 (COX-2) mRNA expression, PI3K/AKT-mediated proliferation and VEGF-mediated angiogenesis and impaired apoptosis. Increased HER4 expression has been observed in all stages of colorectal carcinogenesis, including adenomas, but not in normal colonic mucosa.[28, 29]

HER3 mutations, detected in up to 11% of CRCs, are associated with malignant transformation *in vitro.*[30] HER4 overexpression was documented in 17% of CRC samples and mutations were detected in 2.9% of CRCs.[28, 31] HER3 and HER4 expression, as well as phosphorylated HER3 and HER4, were associated with worse prognosis.[32]

Preclinical experiments suggest that targeting HER3 ant the HRG/HER3 pathway can have therapeutic implications including mediating resistance to cetuximab (an anti-EGFR monoclonal antibody) and vemurafenib (a BRAF inhibitor) treatments. Anti-HER3 antibodies or genetic silencing of the *HER3* gene reduces colon cancer cell proliferation, migration, and invasion.[33] HRGs and the heterodimer HER3/HER2 mediate the development of resistance to cetuximab in preclinical models. In metastatic CRC, patients with high amphiregulin and low HRG plasma levels have higher response rates to cetuximab-based therapies.[34] In BRAF-V600E mutant colon cancer stem cells (CSCs), HER3/Neuregulin-1β induces cellular proliferation and drug resistance to vemurafenib.[35]

### Non-small cell lung cancer (NSCLC)

HER2 and HER3 expression were found in 7% and 32% of resected NSCLC tumors, respectively.[36] Neurotensin upregulation also occurs in 60% of NSCLCs and positively correlates with increased HER3 and HER2 expression.[37] Despite the lack of association between HRGs expression and prognosis in NSCLC, gene fusions involving NRG1 have been identified as drivers of NSCLCs (e.g. *VAMP2-NRG1*).[38, 39] The CD74-NRG1 gene fusion produces an EGF-like domain of NRG1 that can activate HER3/HER2.[40] In preclinical models, residual cells after chemotherapy have increased HRG1 expression and autocrine HER3 and HER4 pathway activation. Also, preclinical data suggest that increased expression of HER3 and NRG1 contribute to resistance to ALK inhibitors.[41–43] Although therapies to block HER2 in NSCLC have had disappointing results in the past, perhaps therapies directed at HRGs, HER3 or HER4 would be more successful. Anti-HER4 treatment has been shown to reduce recurrence after cessation of chemotherapy in the experimental scenario.[44]

### Head and neck squamous cell carcinoma (HNSCC)

HNSCCs display one of the highest expression levels of HRG among different cancers with 40% of specimens expressing high levels of HRGs. HRGs mediate proliferation and invasion and are associated with worse prognosis.[45, 46] HER3 expression, present in 8.8% of HNSCCs, correlates with lower OS.[47] Trop2, a transmembrane protein that forms a complex with NRG-1 in the cytosol, reduces the amount of available NRG-1 available to participate in binding to HERs and plays an important role in regulating the HRG/HER3 pathway in HNSCC.[48, 49] HRG expression is higher in recurrent tumors than in primary tumors suggesting a role in treatment resistance.[50] *NRG1* overexpression is associated with primary resistance to cisplatin, and siRNA-suppression of NRG1 reverses this effect.[51] Furthermore, increased NRG1 mRNA predicts response to cetuximab *in vitro.*[52] These results have fostered interest in targeting this pathway for HNSCC treatment. *In vitro*, HNSCCs respond to anti-HER3 antibodies, with increased response in Trop2 expressing cells.[49] Wilson et al. showed that a subset of HNSCC cell lines respond to lapatinib, a tyrosine kinase inhibitor of EGFR and HER2. Increased NRG1 and phosphorylated HER3 levels were associated with increased lapatinib sensitivity.[53]

### Prostate cancer

NRG-4, HER3 and HER4 expression have been documented in a subset of prostrate cancers.[54] Prostate cancers that overexpress HER3 depend on its expression for malignant progression.[55] In prostate cancer, unlike in many other cancer types, high HRG expression is associated with favorable outcomes. In a cohort of 357 hormone-naïve prostate cancers, high membranous HRG expression was associated with better outcomes (increased time to relapse and OS) and fell significantly in post-relapse specimens.[56]

HRG/HER signaling in prostate cancer is modulated by EBP-1, a HER3-binding protein, that reduces HRG-induced tumor growth and represses androgen receptors expression. EBP-1 levels are decreased in prostate cancer and restoring EBP-1 levels in a prostate cancer animal model decreases tumorigenicity.[57] EBP-1 expression may be decreased by post-transcriptional up-regulation of androgen receptors.[58]

The effect of HRGs on prostate cancer is hormone dependent. HRGs inhibit proliferation in hormone-naïve cells,, whereas HRGs increase tumor proliferation in castration-resistant disease. Exposing hormone-naïve prostate cancer cells to HRGs reduces aneuploidy and proliferation.[59] On the other hand, in androgen-independent prostate cancer, activation of HER2/HER3 increases androgen receptor transactivation and tumor growth.[60]

### Ovarian cancer

HER3 and HER4 are expressed in several different types of ovarian cancer. In ovarian epithelial adenocarcinomas, HER3 was detected in 53.4% of samples.[61] HER4 was detected in 89-95% of serous cystadenocarcinomas.[62] In granulosa cell tumors, HER4 expression was higher than HER2 or HER3. NRG-1α and NRG-1β were expressed in 87% and 77% of tumors, respectively, and were shown to regulate growth of tumor cell lines *in vitro.*[63]

The HRG/HER3 pathway has been implicated in resistance of ovarian epithelial adenocarcinomas to chemotherapy and targeted therapies. In doxorubicin-resistant epithelial carcinoma cells, an anti-apoptotic signaling pathway depends on HER3 ligands, the metalloproteinase ADAM17, and HER2.[64] HER3 is downregulated in patients with poor or no response to chemotherapy.[65] Conversely, HER3 overexpression has been associated with acquired resistance to trastuzumab in ovarian epithelial adenocarcinomas.[66] In a phase II trial of patients with platinum-resistant ovarian epithelial carcinoma, low HER3 mRNA expression predicted response to gemcitabine plus an anti-HER2 antibody (pertuzumab).[67, 68] These results suggest therapeutic potential of the HRG/HER pathway in ovarian cancer treatment.

### Breast cancer

HER3 is overexpressed in approximately 50% of breast cancers and confers a worse prognosis.[69–72] In HER2-amplified breast cancers, HER3 is the preferential partner for dimer formation.[73, 74] HER3 mutations are common in lobular invasive breast cancer.[75] HER4 has been reported as downregulated in 18-75% of breast cancers and upregulated in 7-29% of cases.[76] HER4 expression and the expression of its intracellular domain correlate with luminal and well-differentiated histology, expression of estrogen and progesterone-receptors, low histological grade, low Ki67, tamoxifen-responsiveness, and possibly better clinical outcomes.[76–79] However, nuclear HER4 is linked to poor outcomes in HER2+ breast cancers. [80]

HRGs through HER3 or HER4-mediated signaling participate in the embryonic development of mammary gland.[81–83] HRGs are expressed in the cytoplasm as well as in the nucleus of human breast cancer cells. There are no differences in *NRG2* expression between HER2-positive and HER2-negative breast cancers. However, *NRG2* median expression is increased up to three-fold in estrogen receptor (ER) and progesterone receptor (PR) negative tumors compared to ER- and PR-positive samples.[84] A different study found expression of HER3 ligands (NRG1 and NRG2) in 39.3% of samples and HER4 ligands (NRG1-4, EREG, BTC, HB-EGF) in 74.1% of samples.[84] Rearrangements in the *NRG1* gene were found in 17 of 382 of breast cancer cases, and an amplicon centromeric to NRG1 was found in 63 of 262 cases and correlated with poor prognosis.[85] NRG expression has been associated with both better and worse prognosis. In breast cancer cell lines, overexpression of HRG induces a more aggressive, hormone-independent phenotype with increased angiogenesis and stemness properties.[86–90] HRG overexpression also promotes cell motility, metastasis, and invasiveness. Furthermore, in breast cancer cells, NRG-2β was shown to promote telomere shortening, inducing chromosomal instability.[91] NRG-1β expression in breast cancer stromal cells correlated with a worse prognosis.[92] Also, NRG-2β and NRG-4 correlated with high-grade tumors.[93] On the other hand, in a cohort of 115 breast cancer patients, NRG-1α expression was present in 84% of samples and correlated with a better prognosis.[94] NRG-3 nuclear staining also correlated with low-grade tumors.[92]

The precise role of HER4 in breast cancer carcinogenesis remains not fully understood. HER4 mediates both protumoral and antiproliferative and proapoptotic signals in breast cancer cells. [76]

HER3 overexpression predicts resistance to trastuzumab, though HER3 expression has not been shown as a predictive factor in combination treatment of trastuzumab and pertuzumab in HER2+ breast cancers.[95] HER2-positive cells also acquire resistance to trastuzumab through increased ADAM10-mediated HRG release.[96] HER3 overexpression also correlates with resistance to lapatinib. HRG expression may predict clinical response to trastuzumab in breast cancer without HER2 amplification and can mediate acquired resistance to lapatinib.[97, 98] NRG-Beta1 mediates trastuzumab emtansine resistance and treatment with pertuzumab circumvents this issue.[99]

Limited data are available for other histologies such as pancreatic cancer, thyroid cancers, small cell lung cancer, bladder cancer and sarcomas.

### TARGETING HEREGULIN-DEPENDENT SIGNALING

**Figure 3:**
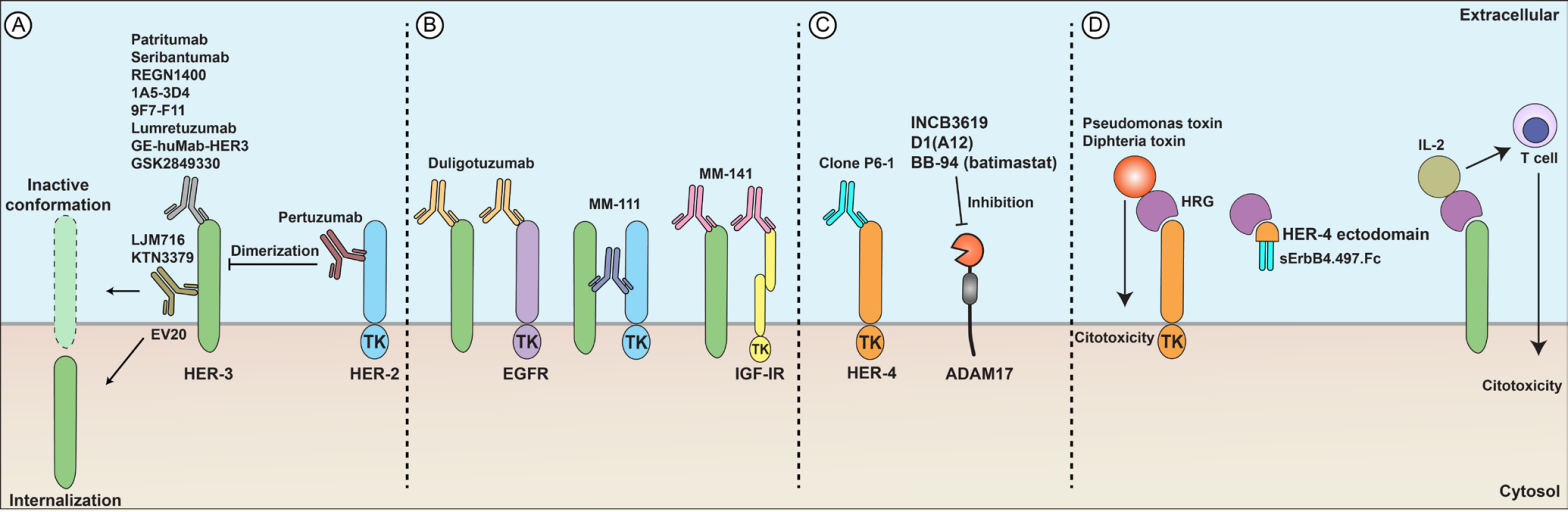
Developmental therapeutics and heregulins (**A**) Anti-HER3 antibodies (patritumab, seribantumab, REGN1400) and anti-HER2 antibodies (pertuzumab) can block receptor dimerization, induce receptor internalization (EV20), or result in an inactive receptor conformation (LJM716 and KTN3379), subsequently impairing activation of downstream pathways and triggering antineoplastic responses. (**B**) Bispecific antibodies directed against HER3 and another growth factor receptor. Duligotuzumab binds to HER3 and EGFR; MM-111 binds to HER3 and HER2; MM-141 binds to HER3 and IGF-IR. (**C**) Anti-HER4 antibodies (clone P6-1) cause growth inhibition of breast cancer cells *in vitro*. ADAM17 inhibitors (batimastat, D1(A12), INCB3619) inhibit liberation of HRG from the cell surface and impair paracrine cell signaling. (**D**) HRG fusion proteins induce direct cytotoxicity (HRG plus Pseudomonas or diphteria toxin) or activate T cells present in the tumor milieu (HRG plus IL-2). HER4 fusion proteins (HER4 ectodomain plus the human IgG Fc) trap HRGs, preventing their binding to functional receptors.

Figure 3 summarizes therapeutics targeting HRG-dependent signaling, including anti-HER3, anti-HER4, bispecific antibodies and HRG fusion proteins.

### Anti-HER3 antibodies

Patritumab (U3-1287 or AMG-888) is a fully humanized antibody to HER3 that impairs HRG binding. In models of HNSCC and lung cancer, patritumab enhanced radiosensitivity.[100] In CRC and NSCLC cell lines and animal models, patritumab reversed resistance to anti-EGFR treatment with cetuximab.[101, 102] In NSCLC cell lines and mouse models, patritumab also improved response to the EGFR tyrosine kinase inhibitor erlotinib. Uveal melanoma cells overcame resistance to MEK inhibitors when treated with patritumab.[103]

The first clinical trial of patritumab (NCT00730470) enrolled 57 patients with advanced solid tumors including 29 patients with CRC and 20 patients with NSCLC. The most commonly observed toxicities were fatigue (21.1%), diarrhea (12.3%), and nausea (10.5%), with only 1.8% incidence of grade 3 or higher adverse events.[104] Table 1 summarizes published and ongoing clinical trials evaluating patritumab.

**Table 1:** Patritumab (U3-1287 or AMG-888; Daiichi-Sankyo) mechanism of action, stage of development and specific study features.

Mechanism of action	Stage	Ref	Identifier	Study features	Results
anti-HER3; impairs ligand-dependent signaling	Phase I	[104]	NCT00730470	advanced solid tumors refractory to standard therapy (the majority CRC or NSCLC).	Safe at 9 to 20 mg/kg every 2 to 3 weeks. AEs: fatigue and diarrhea.
	Phase I	[178]	NCT01957280	tested the safety and pharmacokinetics of a new patritumab formulation in patients with solid tumors	Safe at loading dose of 18 mg/kg and maintenance dose of 9 mg/kg; AEs: diarrhea; no HAHA formation
	Phase Ib/Phase II	[179]	NCT01512199	HER2+ metastatic breast cancer; combination with paclitaxel and trastuzumab	Safe at 9 and 18 mg/kg; no dose-limiting toxicities observed; AEs: diarrhea, alopecia and leukopenia
	Phase I	-	NCT02350712	HNSCCs; combination with cetuximab and platinum containing therapy	completed, not published
	Phase II	[108]	NCT02633800	HNSCCs; combination with cetuximab and platinum containing therapy	ongoing
	Phase I/Phase II	[106]	NCT01211483	platinum-resistant EGFR WT advanced or metastatic NSCLC; combination with erlotinib;	improved PFS in HRG-high, but not in the intention-to-treat population; AEs: rash and diarrhea
	Phase III	-	NCT02134015	platinum-resistant EGFR WT advanced or metastatic NSCLC; combination with erlotinib; HER3-Lung study	ongoing

AEs: reported adverse events; HAHA: human-anti-human antibodies; CRC: colorectal cancer; NSCL: non small cell lung cancer; HNSCC: head and neck squamous cell carcinoma; HRG: heregulins; PFS: progression free survival.

Patritumab was also evaluated in combination with erlotinib for NSCLC, with cetuximab and platinum-based chemotherapy for HNSCC, and with trastuzumab and paclitaxel for HER2 positive breast cancer. A phase I study enrolled 24 Japanese patients with NSCLC after progression on first-line chemotherapy. The patients were treated with patritumab 9 mg/kg and 18 mg/kg every 3 weeks in combination with erlotinib with no grade 3 toxicities.[105] The phase II HERALD trial randomized patients with platinum-resistant, EGFR wild-type, advanced or metastatic NSCLC to erlotinib with high dose patritumab, low dose patritumab or placebo. The subgroup of high HRG expressing tumors showed increased PFS and has supported an ongoing phase III trial (NCT02134015).[106, 107] Phase I (NCT02350712) and phase II (NCT02633800) studies are currently testing the combination of patritumab with cetuximab and platinum-based therapy for HNSCC.[108] A phase Ib/II trial is also evaluating the combination of patritumab, trastuzumab and paclitaxel in first-line treatment of HER2-amplified breast cancers (NCT01512199).

Seribantumab (MM-121 or SAR256212) is a fully human monoclonal antibody targeting HER3.[109] In preclinical models of lung cancer and HNSCC, seribantumab decreased HER3 phosphorylation.[109, 110] A preclinical animal model of ovarian cancer treated with seribantumab also showed decreased tumor growth.[111]

When combined with seribantumab, the EGFR targeted therapies gefitinib, erlotinib and cetuximab have shown enhanced and more sustained activity in preclinical models of NSCLC, pancreatic ductal adenocarcinoma and HNSCC. An EGFR mutant lung cancer cell line was re-sensitized to gefitinib by seribantumab.[109] A mouse model of lung cancer treated with cetuximab and seribantumab showed a durable response compared to cetuximab alone where resistance developed rapidly.[109] In cell lines and animal models of HNSCC, seribantumab combined with cetuximab showed more potent cell and tumor growth suppression by inhibiting activation of HER3, EGFR, PI3K/AKT and ERK.[110] Cetuximab-resistant HNSCC models were found to have upregulation of HER3 and were more effectively treated with combined cetuximab and seribantumab than either antibody alone.[112] In HER2+ breast cancer cell lines, seribatumab enhanced the effect of paclitaxel and delayed the onset of resistance to and restored sensitivity to the aromatase inhibitor letrozole.[113, 114] Seribatumab also showed significant anti-tumor activity in trastuzumab-resistant HER2+ breast cancer cell lines and animal models.[115]

Phase I trials showed that seribantumab has a favorable toxicity profile as a single agent and in combination with chemotherapy or anti-EGFR and PI3K inhibitors.[116–118] A phase II trial evaluated the combination of exemestane +/- seribantumab in post-menopausal women with advanced ER- and/or PR-positive HER2-negative breast cancer (NCT01151046). The trial showed no significant effect on the primary outcome of PFS, but suggested a possible increase in OS, specifically in patients with HRG-positive tumors.[119] A randomized phase II trial (NCT01421472) studied neoadjuvant paclitaxel +/- seribantumab prior to doxorubicin and cyclophosphamide for locally advanced hormone-receptor-positive and triple negative breast cancer. Samples were evaluated for pathological compete response. Overall, the addition of seribantumab showed no significant benefit, but there was a trend toward higher pathologic complete response (10.6%, 95% CI [5.2%, 20.3%] vs. 3.3%, 95% CI [0.6%, 16.7%]) in patients with hormone-receptor-positive breast cancer, but not in patients with triple-negative tumors.[120]

A phase II trial (NCT01447706) evaluating paclitaxel +/- seribantumab in platinum-resistant ovarian cancer demonstrated improved PFS in tumors positive for HRG, betacellulin (an EGFR ligand), HER2, HER3, or EGFR.[121] A phase II trial of erlotinib +/- seribantumab in patients with platinum-resistant, TKI-naïve, wild-type EGFR NSCLC showed no PFS or OS benefit.[122] The phase II SHERLOC trial of chemotherapy (docetaxel or pemetrexed) +/- seribantumab in heregulin positive NSCLC is ongoing (NCT02387216). Despite encouraging results in preclinical models using seribantumab, phase II studies published to date have not shown clinical benefit in general patient populations. However, subgroup analyses from breast and ovarian cancer trials suggest that improving patient selection (i.e. HRGs positive tumors) may translate into significant results but will require confirmation in larger cohorts as well as validation of methods to measure HRGs expression in distinct clinical settings. Additional studies targeting the HRG pathway using seribantumab in this specific population are ongoing in other diseases, as depicted in Table 2.

**Table 2:** Seribantumab (MM-121; Merrimack) mechanism of action, stage of development and specific study features.

Mechanism of action	Stage	Ref	Identifier	Study features	Results
anti-HER3; impairs ligand-dependent signaling	Phase I	[117]	NCT01451632	advanced solid tumors; with cetuximab +/- irinotecan	AEs: diarrhea, hypokalemia, nausea, fatigue, rash
	Phase I	-	NCT01436565	in advanced solid tumors; with anti-PI3K (piralarisib)	completed, not published
	Phase I	-	NCT01209195	advanced gynecologic or breast cancer; with paclitaxel	completed, not published
	Phase I	-	NCT02538627	mCRC, NSCLC, HNSCC; with MM-151 (anti-EGFR)	ongoing
	Phase I	-	NCT00734305	advanced refractory solid tumors; alone	ongoing
	Phase I	[116]	NCT01447225	advanced solid tumors; with chemotherapy	AEs: diarrhea, nausea, fatigue, anemia, hypokalemia, vomiting.
	Phase I/II	[122]	NCT00994123	platinum-resistant NSCLC; with erlotinib	MM-121 was tolerated at 20 mg/kg every other week; combination was not effective in prolonging PFS
	Phase IIR	-	NCT02387216	heregulin positive NSCLC; with chemotherapy;	ongoing
	Phase II	[180]	NCT01151046	hormone receptor-positive HER2-negative advanced breast cancer; with exemestane	Combination of MM-121 and exemestane did not prolong PFS; prolonged PFS in patients positive for two of pre-specified biomarkers (HRG, betacellulin, EGFR, HER2 and HER3),
	Phase II	[121]	NCT01447706	platinum-resistant EOC; with paclitaxel	Combination with MM-121 did not prolong PFS; prolonged PFS in biomarker-guided population
	Phase IIR	[120]	NCT01421472	TNBC or HR+ breast cancer; with paclitaxel; preoperative setting	MM-121 increased complete pathologic response in HR+ group (10.6% vs 3.3%)

AEs: adverse events; CRC: colorectal cancer; NSCL: non small cell lung cancer; HNSCC: head and neck squamous cell carcinoma; EOC: epithelial ovarian cancer; TNBC: triple negative breast cancer; HRG: heregulins; PFS: progression free survival.

LJM716 (Table 3) is an anti-HER3 antibody that traps HER3 in an inactive conformation and inhibits ligand-dependent and independent activation.[123] In HER2+ breast and gastric cancer cell lines and xenografts, decreased growth was seen after treatment with LJM716 alone and in combination with lapatinib/trastuzumab or alpelisib, a PI3K inhibitor.[124] Phase I studies have shown that LJM716 is safe and well tolerated in advanced solid tumors (NCT01598077, NCT01911936).[125, 126] A phase I study (NCT01602406) of LJM716 in combination with trastuzumab in patients with advanced HER2-positive breast or gastric cancer demonstrated safety and provided preliminary efficacy results with 40% of patients achieving stable disease.[127] Additional trials are investigating the safety of LJM716 in combination with PI3K inhibitors and trastuzumab (NCT01822613, NCT02167854).[128]

**Table 3:** LJM716 and KTN3379 mechanism of action, stage of development and specific study features

Antibody	Mechanism of action	Stage	Ref	Identifier	Study features	Results
LJM716 *Novartis*	anti-HER3; impairs ligand-dependent and ligand-independent signaling	Phase I	[181]	NCT01911936	advanced solid tumors; in Japanese patients	well tolerated at 10 to 40 mg/kg once weekly; AEs: diarrhea, fatigue, stomatitis, pyrexia and paronychia
		Phase I	[125]	NCT01598077	advanced or metastatic HNSCC or HER2+ breast or gastric cancer	Well tolerated in doses up to 40 mg/kg once weekly; AEs: diarrhea, hypokalemia, asthenia, chills, infusion-related reactions
		Phase I	-	NCT02143622	platinum refractory advanced HNSCC; in combination with cetuximab	study was terminated prior to enrollment
		Phase I	[127]	NCT01602406	advanced HER2-positive breast or gastric cancer; combination with trastuzumab	Safe at 3 to 40 mg/kg once weekly; AEs: diarrhea, nausea, fatigue and chills
		Phase I	[128]	NCT02167854	advanced HER2-positive breast cancer; combination with BYL719 (PI3K inhibitor) and trastuzumab	Safe at 20 mg/kg once weekly in this combination; gastrointestinal and metabolic toxicities limited drug delivery
		Phase I/Phase II	-	NCT01822613	refractory esophageal squamous cell carcinoma; combined with BYL719 (PI3K inhibitor)	completed, not published
KTN3379 Kolltan	anti-HER3; impairs ligand-dependent and ligand-independent signaling	Phase I	[133]	NCT02014909	refractory advanced SCCHN, CRC, HNSCC, melanoma and HER2+ breast cancer; alone or combined with cetuximab, erlotinib, vemurafenib and trastuzumab	Safe at 5 to 20 mg/kg every 3 weeks; main side effects were diarrhea, mucositis and rash
		Phase I	-	NCT02473731	window-of-opportunity study to evaluate downstream molecular pathways to identify potential tumor response and resistance mechanisms in HNSCC	ongoing

AEs: adverse events; CRC: colorectal cancer; NSCL: non small cell lung cancer; HNSCC: head and neck squamous cell carcinoma; HRG: heregulins

REGN1400, (Table 4) an anti-HER3 antibody, increased sensitivity to anti-EGFR treatment in HNSCC in xenografts models.[129] A clinical trial showed that REGN1400 is safe and well tolerated alone or in combination with erlotinib or cetuximab in patients with advanced CRC, HNSCC and NSCLC (NCT01727869).[130]

**Table 4:** Duligotuzumab (MEHD7495A), REGN1400, and GSK2849330 mechanism of action, stage of development and specific study features

Antibody	Mechanism of action	Stage	Ref	Identifier	Study features	Results
Duligotuzumab (MEHD7495A) *Genentech Inc*	anti-HER3 and anti-EGFR bispecific antibody	Phase I	[153]	NCT01207323	advanced solid tumors	well tolerated at 1 to 30 mg/kg every 2 weeks; AEs: diarrhea, nausea, headache, fever; 2 patients (HNSCCs) had radiologic responses
		Phase IIR	[155]	NCT01577173	platinum-refractory HNSCC	Did not improve outcomes in comparison to cetuximab
		Phase IIR	[156]	NCT01652482	mCRC after progression to oxaliplatin-based chemotherapy	FOLFIRI + MEHD7495A did not improve outcomes in comparison to FOLFIRI + cetuximab
		Phase Ib	[154]	NCT01911598	HNSCC; in combination with chemotherapy	Safe at 1650 mg/kg every 3 weeks; main side effects were diarrhea, neutropenia and fatigue
		Phase I	-	NCT01986166	locally advanced or mCRC with mutant KRAS; in combination with cobimetinib	completed, not published
REGN1400 *Regeneron*	anti-HER3; impairs ligand-dependent signaling	Phase I	[130]	NCT01727869	patients with advanced NSCLC, CRC or HNSCC; alone or in combination with erlotinib or cetuximab	well tolerated at 3, 10 and 20 mg/kg every 2 weeks; AEs: rash, diarrhea, nausea, hypomagnesemia
GSK2849330 *GlaxoSmithKline*	glycoengineered anti-HER3 antibody with enhanced ADCC and CDC activities	Phase I	-	NCT01966445	advanced HER3-positive solid tumors	ongoing
		Phase I	-	NCT02345174	advanced HER-3 expressing solid tumors; to evaluate the uptake of Zirconium-89-labeled-GSK2849330	completed, not published

AEs: adverse events; CRC: colorectal cancer; NSCL: non small cell lung cancer; HNSCC: head and neck squamous cell carcinoma; HRG: heregulins

KTN3379 (Table 3) is an anti-HER3 monoclonal antibody that binds to an epitope that locks HER3 in its inactive conformation and inhibits both ligand-dependent and ligand-independent signaling.[131] Preclinical data show that the anti-tumor effects of KTN3379 were attenuated in PTEN-knockdown tumor cell lines.[132] A phase I study (NCT02014909) has proven safety of KTN3379 alone or in combination with cetuximab, erlotinib, vemurafenib or trastuzumab in patients with advanced solid tumors.[133]

Av-203 (Table 5) is an IgG1k humanized anti-HER3 monoclonal antibody that inhibits tumor growth in human cancer models with the level of NRG1 expression predictive of response.[134] A phase I clinical trial in patients with advanced solid tumors, including CRC, NSCLC, and HNSCC, showed that Av-203 is safe and well tolerated below a maximum dose of 20 mg/kg every 2 weeks.[135] One patient with NSCLC had a partial response.

**Table 5:** Lumretuzumab (RG7166 or RO5479599) and AV-203 mechanism of action, stage of development and specific study features

Antibody	Mechanism of action	Stage	Ref	Identifier	Study features	Results
Lumretuzumab (RG7116 or RO5479599) *Regeneron*	anti-HER3; impairs ligand-dependent signaling; downregulates membranous HER3; potentiates ADCC	Phase I	[138]	NCT01482377	HER3-positive advanced solid tumors	well tolerated at 100 to 2,000 mg every 2 weeks; common toxicities were diarrhea, fatigue, decreased appetite; 10 patients had stable disease
		Phase I	[182]	NCT02204345	advanced NSCLC of squamous histology; in combination with carboplatin and paclitaxel	Safe at 800 mg every 2 weeks; AEs: diarrhea, asthenia, neurotoxicity; 3 patients with high HRG mRNA expression had partial responses
		Phase I	-	NCT01918254	HER3&HER2-positive metastatic breast cancer; in combination with paclitaxel and pertuzumab	completed, not published.
AV-203 *AVEO*	anti-HER3; impairs ligand-dependent and ligand-independent signaling	Phase I	[135]	NCT01603979	metastatic or advanced solid tumors	Safe at 2 to 20 mg/kg every 2 weeks; AEs: diarrhea, decreased appetite, hypokalemia, dry skin, hypomagnesemia and pruritus; 1 patient with squamous cell NSCLC had a partial response

AEs: adverse events; NSCL: non small cell lung cancer; HRG: heregulins

Lumretuzumab (RG7116, RO5479599) is a glycoengineered anti-HER3 antibody, which impairs HRG binding to HER3 and induces antibody dependent cytotoxicity in preclinical models.[136] Efficacy has been demonstrated in experimental models of HNSCC.[137] A recent phase I study proved the safety of RG7116 in patients with HER3-positive advanced solid tumors and showed stable disease in 21.3% or patients and partial response in 23.7% of patients (Table 5).[138]

There are at least nine other anti-HER3 antibodies in development. 1A5-3D4 is an anti-HER3 antibody that in combination with trastuzumab has shown tumor size reduction in preclinical gastric cancer xenografts.[139] 9F7-F11 is an anti-HER3 antibody that induces apoptosis in cell lines by increasing HER3 ubiquitination and degradation through JNK-dependent ITCH/AIP4 activation. In pancreatic cancer xenografts, 9F7-F11 induced tumor regression.[140] GE-huMab-HER3 is a glycoengineered anti-HER3 antibody that enhances antibody-dependent cell-mediated cytotoxicity and increases antitumor effect compared to the non-glycoengineered variant of the antibody WT-huMab-HER3.[141] GSK2849330 is a glycoengineered anti-HER3 monoclonal antibody that increases both complement-mediated and antibody-dependent cell-mediated cytotoxicity (CDC).[142] Phase I studies of GSK2849330 are ongoing (NCT01966445, NCT02345174) (Table 4). EV20 is an humanized anti-HER3 antibody, which interferes with ligand-dependent and independent signaling and causes internalization of HER3 in several cancer cell lines.[143] EV20 reversed resistance to vemurafenib in BRAF-V600E mutant colon cancer stem cells.[35] HuHER3-8 is an anti-HER3 antibody that, when combined with BRAF inhibitors, reduces tumor growth in melanoma xenografts with WT BRAF [144] or V600E mutated BRAF. [145] LMAb3 is an anti-HER3 antibody that can reverse HRG-mediated acquired resistance to anti-HER2 agents in ovarian cancer.[146] SGP1 is an anti-HER3 antibody that impairs HRG binding to HER3 and enhances antitumor effects when combined with trastuzumab.[147] Ab6 is an anti-HER3 antibody that, when combined with trastuzumab, reverses resistance to PI3K inhibitors in prostate cancer cells.[148]

### Anti-HER4 antibodies

Recently, Okazaki et al. showed that an anti-HER4 antibody (clone P6-1) resulted in growth inhibition of breast cancer cells in a three-dimensional extracellular matrix culture system.[19]

### Bispecific antibodies

Antibodies have been developed that simultaneously target HER3 and another receptor including EGFR, HER2 or IGF-1R. Duligotuzumab (MEHD7945A) is a bispecific anti-HER3 and anti-EGFR antibody. In preclinical models, it was more effective at inhibiting EGFR and HER3 mediated signaling than monospecific anti-HER3 antibodies.[149] In animal models, duligotuzumab increased radiosensitivity in NSCLC and HNSCC, overcame resistance to EGFR inhibitors in HNSCC, and increased response to PI3K inhibitors in triple negative breast cancer.[150–152] A phase I clinical trial (NCT01207323) of single agent duligotuzumab in locally advanced or metastatic refractory epithelial tumors documented its safety profile and showed partial responses in 2 of 12 patients with HNSCC that expressed high levels of HRGs.[153] A phase Ib trial (NCT01911598) showed that duligotuzumab is safe in combination with chemotherapy in patients with recurrent or metastatic HNSCC.[154] A phase II study (NCT01577173) did not show improved outcomes in platium-refractory HNSCCs treated with duligotuzumab compared to those treated with cetuximab.[155] A phase II trial (NCT01652482) of patients with KRAS exon 2 wild-type mCRC with progression of disease after oxaliplatin-containing chemotherapy compared duligotuzumab plus FOLFIRI to cetuximab plus FOLFIRI with no significant improvement in clinical outcomes.[156] Table 4 compiles published and ongoing studies investigating duligotuzumab.

MM-111 is a bispecific antibody that forms a trimeric complex with HER3 and HER2, resulting in inhibition of HER3 signaling. In preclinical models of HER2 overexpressing tumors it has shown anti-tumor activity.[157] In phase I trials (NCT01097460, NCT01304784, NCT00911898), MM-111 was safe and well tolerated.[158] A phase II trial NCT01774851), however, did not show any benefit in combining MM-111 with paclitaxel plus trastuzumab in HER2 expressing gastroesophageal cancers (Table 6). These results were possibly related to lower than anticipated HRG expression in the patient population.[159] A possible strategy to improve clinical benefits of dual targeting EGFR and HER3 is to limit inclusion to tumors with high expression of HER3 and/or HRGs.

**Table 6:** MM-111 and MM-141 mechanism of action, stage of development and specific study features

Antibody	Mechanism of action	Stage	Ref	Identifier	Study features	Results
MM-111 *Merrimack Pharmaceuticals*	binds to HER3 and HER2	Phase I	-	NCT01097460	advanced HER2 and HRG-positive breast cancer; combination with trastuzumab	completed, not published
		Phase I	[158]	NCT01304784	advanced HER2-positive cancers; combination with multiple treatments	recommended phase 2 doses: 20 mg/kg once a week and 40 mg/kg every 3 weeks; AEs: anemia, diarrhea, stomatitis, hypokalemia
		Phase I	-	NCT00911898	advanced HER2 and HRG-positive cancers; monotherapy	completed, not published
		Phase II	[159]	NCT01774851	HER2-positive carcinomas of the distal esophagus, gastroesophageal junction and stomach; combination with paclitaxel and trastuzumab	No significant improvement of PFS or OS
MM-141 *Merrimack Pharmaceuticals*	Binds to HER3 and IGF-IR, preventing HRG and IFG signaling	Phase I	[164]	NCT01733004	advanced and refractory solid tumors	Safe at 6, 12 or 20 mg/kg once a week, or at biweekly 40 mg/kg; main toxicities were vomiting, fatigue and abdominal pain
		Phase II	-	NCT02399137	metastatic pancreatic adenocarcinoma; first-line treatment combined with gemcitabine and nab-paclitaxel	ongoing

AEs: adverse events; HRG: heregulins

MM-141 is a bispecific tetravalent antibody to HER3 and IGF-1R. IGF-1R signaling activates the PI3K/AKT survival pathway and is involved in resistance to EGFR and HER2 inhibitors.[160, 161] Likewise, resistance to anti-IGF-1R therapies is mediated by HER3/HRG signaling.[162] In fact, MM-141 overcame resistance to anti-IGF-1R therapies and improved anti-tumor responses in preclinical models.[163] The safety profile of MM-141 was established in a phase I trial and an ongoing study is evaluating combination with gemcitabine and nab-paclitaxel in metastatic pancreatic cancer (NCT02399137) (Table 6).[164]

### Anti-metalloproteinase agents

Anti-metalloproteinase agents have shown promise in the preclinical setting. INCB3619, a specific ADAM17 metalloproteinase inhibitor, blocked HER3 signaling in gefitinib-resistant NSCLC cell lines.[165] D1(A12), another ADAM17 inhibitor, decreased pro-tumor signaling in HNSCC and ovarian cancer models.[48, 166] Batimastat (BB-94), a broad spectrum anti-metalloproteinase, prevented HER3 phosphorylation and Erk activation in fulvestrant-resistant breast cancer cell lines in a mechanism independent of ADAM17, suggesting potential therapeutic application in breast cancer.[167] Early clinical trials to evaluate safety and efficacy of these agents are expected.

### Heregulin fusion proteins

Chimeric HRG-toxin fusion proteins consisting of a HRG, or at least the EGF-like extracellular binding domain, attached to Pseudomonas exotoxin A or diphtheria toxin have shown cytotoxic activity against human breast tumor cell lines expressing HER3 and/or HER4.[168, 169] HER4 expression may be necessary for HRG-toxin fusion protein cytotoxicity.[170, 171] Yang et al. tested eight chimeric toxins composed of the extracellular EGF-like domains of four HRG isoforms combined with truncated Pseudomonas exotoxin (PE38KDEL) and found that the EGF-like domain of HRG13 and HRGbeta2 demonstrated the highest cytotoxic activity.[170]

Bivalent HRG ligands composed of two linked NRG or EGF moieties have been engineered to increase HER homodimer formation and prevent the formation of HER3/HER2 heterodimers. *In vitro*, HRG-HRG fusion proteins inhibit migration and proliferation and induce apoptosis of cancer cells.[172] Other fusion proteins composed of HRG attached to IL-2 or CD3 have been developed to bring T cells into proximity of tumor cells expressing HERs. Lustgarten et al. showed that HRG-IL-2 fusions proteins can redirect non-tumor specific cytotoxic lymphocytes to the tumor site and induce lysis of tumor cells in a non-MHC-restricted manner.[173] The fusion protein of HRG attached to the CD3 zeta-chain causes T lymphocytes to recognize and attack breast cancer cells overexpressing HER3 and HER4.[174, 175]

A fusion protein (sErbB4.497.Fc) comprised of the HER4 ectodomain fused to the human IgG Fc constant region was created and is able to efficiently bind to betacellulin and HRG-beta1 with high affinity. When HRG was bound to the fusion protein receptor, EGFR phosphorylation and downstream signaling were inhibited. The fusion receptor also inhibited proliferation of breast cancer cell lines and had a modest effect on tumor growth in a mouse model.[176]

### Silencing of HER3 expression

A locked nucleic acid-based HER3 antisense oligonucleotide (EZN-3920) decreased HER3 mRNA expression and tumor growth in breast and lung cancer models.[177] Table 7 summarizes the proposed mechanism of action of these promising novel strategies targeting HRG-mediated pathways under preclinical development.

**Table 7:** Preclinical development of novel strategies targeting heregulin-mediated pathways

Class	Drugs	Proposed mechanism of action
metalloproteinase inhibitors	INCB3619 D1(A12)	ADAM17 (TACE) inhibitor; prevents shedding of HRG and consequent binding to target receptors
	Batimastat (BB-94)	Broad spectrum metalloproteinase inhibitor; prevents shedding of HRG and consequent binding to target receptors
Heregulin fusions proteins	HAR-TX beta 2	heregulin-beta 2 fused to a binding-defective form of Pseudomonas exotoxin A; induce cell cytotoxicity preferentially in HER4-positive vells
	DT(389)hrg	a chimera of diphtheria toxin and EGF-like domain of heregulin beta1 induced cytotoxicity against HER3 and/or HER4-expressing cell lines
	Heregulin-IL2 fusions protein	can redirect non-tumor specific cytotoxic lymphocytes to the tumor site and induce lysis of tumor cells in a non-MHC-restricted manner
Chimeric antigen receptor (CAR)-T cells	Heregulin-zeta T cell receptor	Increases recognition and elimination of target cells (HER3 and HER4-positive cells)
Soluble heregulin receptors	sErbB4.497.Fc	A fusion protein constituted of the ErbB4 ectodomain fused to the human IgG Fc constant region; traps heregulin and betacellulin preventing receptor activation
HER3 antisense oligonucleotide	EZN-3920	downmodulate HER3 expression
anti-HER4 antibody	clone P6-1	reduced MCF7 tumor growth; reduces HER4 activation possibly through impairing ligand binding

## CONCLUSIONS

Activation of the HRG signaling network is relevant to various malignancies and is associated with worse clinical outcomes. The presence of multiple types of HRGs and receptors combined with complex downstream signaling makes the translation of preclinical discoveries into effective therapeutics challenging. Nevertheless, several molecules targeting HRG-related pathways are in clinical development and have shown favorable toxicity profiles and preliminary efficacy in several malignancies, alone and in combination with other therapies. Given the extensive crosstalk of HRG-dependent signaling with pivotal pathways regulating both carcinogenesis and treatment resistance, the future of HRG-based therapies may rely on combination with other targeted agents or sequencing treatments guided by emergence of resistance. There is also potential for combination with immunotherapy. The HRG fusion proteins involving IL-2 or CD3 create an interface between the HRG/HER pathway and the immune system, building the foundation for combinations with checkpoint inhibitors and better understanding of the role of HRG signaling in the anti-tumor immune response. A promising strategy is to reverse resistance to anti-HER2 treatment through targeting HRG-dependent pathways in biomarker-selected individuals. Further validation of biomarkers reflecting tumor dependence on the HRG/HER pathway will assist with improved patient selection for treatments targeting this pathway. For example, the promising results of the anti-HER3 antibody seribantumab in subgroups of breast and ovarian cancers with high expression of heregulin highlight the critical importance of validated biomarkers. A promising strategy is to targeting HER3 to reverse resistance to anti-HER2 treatment in biomarker-selected individuals. Between the demonstrated involvement of the HRG/HER pathway in multiple cancer types, the proven efficacy of therapies targeting this pathway, and the extensive list of novel agents in development, it is expected that therapies targeting HRG, HER3, and HER4 will have a meaningful clinical impact on cancer treatment.

## Abbreviations

ADAM17: ADAM metallopeptidase domain 17
AKT: protein kinase B
APIP: Apaf-1-interacting protein
BRAF: B-Raf and v-Raf murine sarcoma viral oncogene homolog B
CSC: cancer stem cell
CRC: colorectal cancer
EBP-1: ErbB3-binding protein 1
EOC: epithelial ovarian carcinoma
EHCCs: extra-hepatic cholangiocarcinomas
ERK: Extracellular Signal-regulated Kinase
GC: gastric cancer
HER: human epithelial receptor
HCV: hepatitis C virus
HNSCCs: head and neck squamous cell carcinoma
HRGs: heregulins
ICD: intracellular domain
IHCCs: intrahepatic cholangiocarcinomas
IL-2: interleukin-2
JAK: janus kinase
JNK: c-Jun N-terminal kinases
MAPK: Mitogen-activated protein kinase
mCRC: metastatic colorectal cancer
MEK: MAPK kinase
MITF: microphtalmia-associated transcription factor
MTOR: mechanistic target of rapamycin
NDF: neu differentiation factor
NEDD4: neural precursor cell expressed developmentally downregulated-4
NSCLC: non small cell lung cancer
NRG: neuregulin
PDA: pancreatic ductal adenocarcinoma
PI3K: phosphatidylinositol-3-kinases
SNP: single nucleotide polymorphism
STAT: Signal Transducer and Activator of Transcription
TACE: tumor necrosing factor alpha converting enzyme
VEGF: Vascular endothelial growth factor
TCGA: The Cancer Genome Atlas
YAP: Yes-associated protein
